# The Post-war Revival of Canadian Planning: Assessing the Impact of the Community Planning Association of Canada

**DOI:** 10.1177/15385132231222853

**Published:** 2024-01-22

**Authors:** David L. A. Gordon, Miranda Virginillo

**Affiliations:** 1 4257 Queen’s University, Kingston, ON, Canada

**Keywords:** community planning, suburbs, post-war reconstruction, Canada

## Abstract

The Community Planning Association of Canada (CPAC) advocated for the re-establishment of planning in post-war Canada. During this period, the federal government set reconstruction objectives, and both Central (now Canada) Mortgage and Housing Corporation (CMHC) and the CPAC were formed. We believe that 1944–1947 was a critical juncture establishing planned suburban development in Canada as a path-dependent process with tremendous momentum into the 21st-century. Using a historical-institutional approach, the role of CMHC and the influence of the CPAC is examined. Analysis relying on extensive archival material demonstrates that the CPAC gave a tremendous push along the path-dependent process of suburbanization.

## Introduction

It is widely acknowledged that Canada is now a ‘suburban nation’ but how did it make the transition from urban to suburban so quickly after World War II?

Canadian planning and housing construction had essentially collapsed in the years leading up to the end of WWII. The *Report of the Subcommittee for Housing and Community Planning*^1^ from the federal government’s Advisory Committee on Reconstruction indicated that there was strong need for planning along with the construction of new homes. Named after the chair of the Committee – Professor Clifford Curtis – the Curtis Report recommended numerous actions that could be taken to ameliorate the problem, including the formation of a federal agency to respond to these urgent needs. This agency was formed in 1946 as the Central (now Canada) Mortgage and Housing Corporation (CMHC) and continues to exist today. CMHC followed through with another of the Curtis Report’s recommendations and formed the Community Planning Association of Canada (CPAC) later in 1946 to foster public understanding, and participation in, community planning.^2^ CPAC produced numerous publications and media that were distributed across Canada to interested members of the public, municipal and provincial officials, architects, engineers, the few practicing planners in Canada at the time, and others. The Association held conferences throughout Canada to discuss the benefits of ‘good’ suburban community planning (among other topics). CPAC was somewhat similar to the American Society of Planning Officials (ASPO) and shared conferences with ASPO in Montreal (1955);^3^ Toronto (1965);^4^ and Vancouver (1975). CPAC operated at national, regional, and local scales across Canada from 1946 until the Association’s eventual collapse in 1978. However, very little research has been conducted on CPAC’s actions, actors and interests in the immediate post-war period.^5^ For example, CPAC is not mentioned in Richard Harris’ *Creeping Conformity*, a leading book on Canadian suburban history.^6^

## Theoretical Framework

The primary theoretical framework of the research program is historical institutionalism with the federal government instigating a path-dependent process regarding the suburbanization of Canada. Filion describes ‘dispersed suburbanism’, as a system of development that was ‘shaped by massive government highway and single-family homeownership programmes’, and that the associated lifestyle and values that come with this form of suburbanization ‘reverberate at a society-wide scale and as such constitute an impediment to societal transformation’.^7^ These statements, along with those made by Harris, support the hypothesis that the federal government aligned itself with corporate developers in the post-war period, during a time when it had access to greater autonomy and resources with regards to industrial and residential development, in order to create an economy centred around suburban living and its associated consumerism.^8^

In their article on path dependence and suburbanization in Halifax, Grant, Filion, and Low state that ‘during the post-war period, federal mortgage insurance and housing and planning guidelines designed to stimulate home-ownership and prosperity influenced building standards and suburban designs, with lingering repercussions on form’.^9^ The relationship between the government and large developers, as well as the federal and provincial governments use of zoning bylaws, distribution of funding, and multiple amendments to the *National Housing Act*, all point to its interest in using the housing industry to bolster the post-war economy and create a new source of financial stability for the country and its citizens. By arguing that the government established a path-dependent process from a point of critical juncture, our research analyzes the decisions of the Canadian federal government and its usage of tools such as community planning institutions, a national mortgage industry, educational institutions, and other checks, balances and incentives to harness the power of the post-war era and set the country on the trajectory it continues on today in becoming a suburban nation.^10^

Current theories of path-dependency are the basis for the institutional analysis of the activities of CMHC and CPAC.^11^ This historical-institutionalist research approach helps untangle shared understandings about community planning, good suburban development practices and the standard operating methods and procedures followed by the actors in the Canadian (sub)urban development processes.^12^

Historical institutionalism (HI) is a research method that puts institutions at the centre of social and political analysis; an approach that is particularly appropriate given CMHC’s pivotal position in the creation of CPAC and Canadian suburban development.^13^ A central insight from the HI approach is that new institutions are often created during periods of crisis, such as the establishment of CMHC as an instrument to assist with the reconstruction of the Canadian economy at the close of the Second World War.

In HI terms, periods of major institutional change are identified as ‘critical junctures’,^14^ when existing structures are not solving patterns such as a housing crisis, and change is less constrained than in the periods of path-dependence that precede and follow them.^15^ This research project considers whether the 1945-55 decade was a critical juncture in Canadian urbanism, when some early decisions (mandatory community planning; facilitating mortgages but avoiding mortgage interest deductibility; privately-developed suburbs rather than publicly-developed new towns) may have set Canada on a suburban path substantially different than the USA or UK.

Sorensen discusses classifying cities as ‘collections of institutionalized property (including public property), produced in particular circumstances and through specific sets of rules’.^16^ Within the context of the historical institutionalist framework as it pertains to planning history, in order to determine whether a critical juncture and path-dependent institutions are present, several elements must be demonstrated:^17^

• A positive feedback effects loop, created by an institution (or institutions), to instigate a specific, long-standing pattern of development;
• Creating a system which prioritizes certain actors/opportunities and their resultant effects on the overall structure being created, and
• Co-evolutionary processes of development between the instigating actors and their partners in maintaining the chosen system.

Other scholars have demonstrated that Canadian suburban development exhibits the characteristics of a path-dependent process.^18^ This article considers whether and how CPAC may have been a central institution that contributed to that path-dependency. In order to demonstrate that this is what occurred in the suburbanization of Canada, the research team looked for evidence in the primary source materials that solidifies the present hypothesis that the strategic partnership between the government, corporate developers and large-scale builders, with the intent of boosting Canadian consumerism and, subsequently, its economy in the post-war years created sets of enduring institutions and community planning approaches that have had continuing impacts upon the processes and patterns of suburbanization in Canada.

The Historical-Institutional method does not expect that institutions are locked into a particular cause of action, but rather that early choice at critical junctures tend to create different evolutionary trajectories of institutional development.^19^ Sorensen^20^ concludes:

“ … The implication for suburban planning histories is that research should focus on the critical junctures when planning regulations for suburban land development were first established, as these shape later trajectories of planning law and suburban development ….”

We adopted Historical Institutionalism as the methodological framework for this project because it was strongly recommended by Sorensen for suburban land development research.^21^ We examined how CMHC and CPAC reinforced these trajectories in the post-war era by publishing planning handbooks, establishing national standards, seeding a network of planning schools, importing planners, and designing and developing lands owned by the federal government. We focused upon the planning and design of suburban communities, extending the work of Harris and others on suburban house design and financial markets.^22^

## Archival Research Methods

The primary methods employed in this research program involved analysis of textual materials from various governmental archives. The research team examined correspondence, planning and policy documents, photographs, maps, and other materials related to the development of Canada’s suburban agenda in the post-war period. The primary source materials helped assess the relationship between individuals residing in urban and suburban spheres, and the actors responsible for developing these spheres, including large-scale developers, government entities, and planners. In addition, we considered the effects that this process had on the continuing development of Canadian housing and suburban communities.

Similarly, our analysis of the documents in their archival settings engaged the question of whether community members were true participants in the consultation process undertaken under the auspices of the CMHC’s community planning initiative, which occurred within the scope of its suburbanization mandate, or whether the feedback sought by the government via subsidiary organizations such as CPAC was merely meant as a tokenistic gesture.

Initially, the research examined resources in the Canadian Centre for Architecture (CCA), the Canadian Housing Information Centre (now the Housing Knowledge Centre) and archives at McGill and Harvard. The Clifford Curtis fond at the Queen’s University Archives allowed us to explore government decision making in the post-war period regarding owner-occupied housing and the use of crown corporations and mortgage incentives to nudge Canadian urban development towards extensive suburbanization. A section of these fonds contains information on housing, economics, and planning, all of which played a part in the work of the Advisory Committee, which subsequently informed the government’s creation of the CMHC. The analysis of the immediate post-war period as a critical juncture leading Canada to become a suburban nation is informed by correspondence from the years during which Curtis chaired the Advisory Committee,^23^ and government reports and papers written on the topics of housing and community planning.

The Humphrey Carver fonds at the CCA provided guidance^24^ into the third phase of the project: examining the fonds of CPAC and CMHC at Library and Archives Canada (LAC).^25^ The CPAC fonds were examined during Summer 2021.^26^

The combination of the CMHC and CPAC holdings at Library and Archives Canada included information on the formation of CPAC, its day-to-day operations and, specifically, CMHC’s role in the creation of CPAC to encourage local jurisdictions to adopt suburban development in planned communities.^27^

## Results

**Figure 1.**
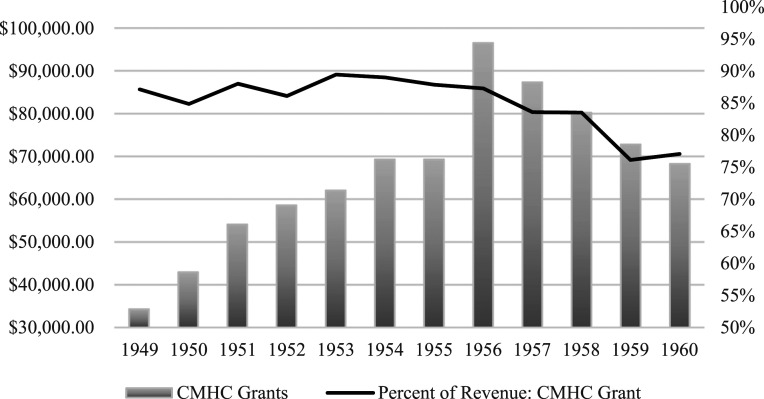
CMHC financial support for CPAC activities, 1949–1960. This graph compares the value (in Canadian dollars) of CMHC grants (left y-axis) and the percent of revenue the CMHC grant accounted for (right y-axis). Note that CMHC’s grants exceeded 75% of the CPAC revenue throughout the post-war period. CPAC never overcame its dependence on federal funding and the national organization collapsed in the 1970s after CMHC’s grants ceased. Data Source: CPAC Annual Reports, CPAC fonds, Library and Archives Canada, MG28 I 14.

**Figure 2.**
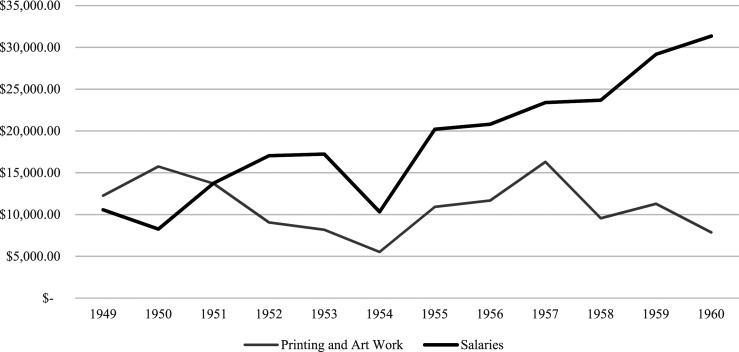
CPAC’s spending on printing & art work vs. salaries. CPAC maintained a robust publishing program in the post-war period, distributing thousands of pamphlets, newsletters and monographs. The cost of staffing the provincial and national offices gradually expanded to take up a much larger proportion of the CPAC’s budget. Data Source: CPAC Annual Reports, CPAC fonds, Library and Archives Canada, MG28 I 14; funds are reported in Canadian dollars.

Examination of CPAC’s financial statements and grant applications submitted to CMHC between 1946 and 1960 reveals that CMHC’s funding was crucial for CPAC to be able to promote community planning across Canada; membership fees and other grants were not enough to support their activities. The CPAC applied for and was awarded CMHC grants that accounted for the vast majority of CPAC’s revenue every year from 1947 into the 1970s. In the post-war period, CMHC provided between 76% and 90% of CPAC’s revenue (Figure 1). CPAC’s two greatest expenses were for publications (Printing and Art Work) and for employees (Salaries) (Figure 2). CPAC employed several full-time staff members in the national office in Ottawa and had employed full-time regional secretaries in British Columbia, Ontario, and the Maritime provinces to coordinate CPAC’s activities. Both the publications and the staff were integral to CPAC’s ability to promote community planning across Canada in the post-war period.

Analysis of the actions, actors and interests of CPAC reveals that CPAC had three main focus areas in the immediate post-war period: education on subdivision and community design principles, adequate supply of qualified planning professionals, and provision of better planning education and enabling legislation across Canada. CPAC used their strongest tools – their regular periodicals^28^ and special publications^29^ – to promote these three areas of interest.

## Publications

**Figure 3.**
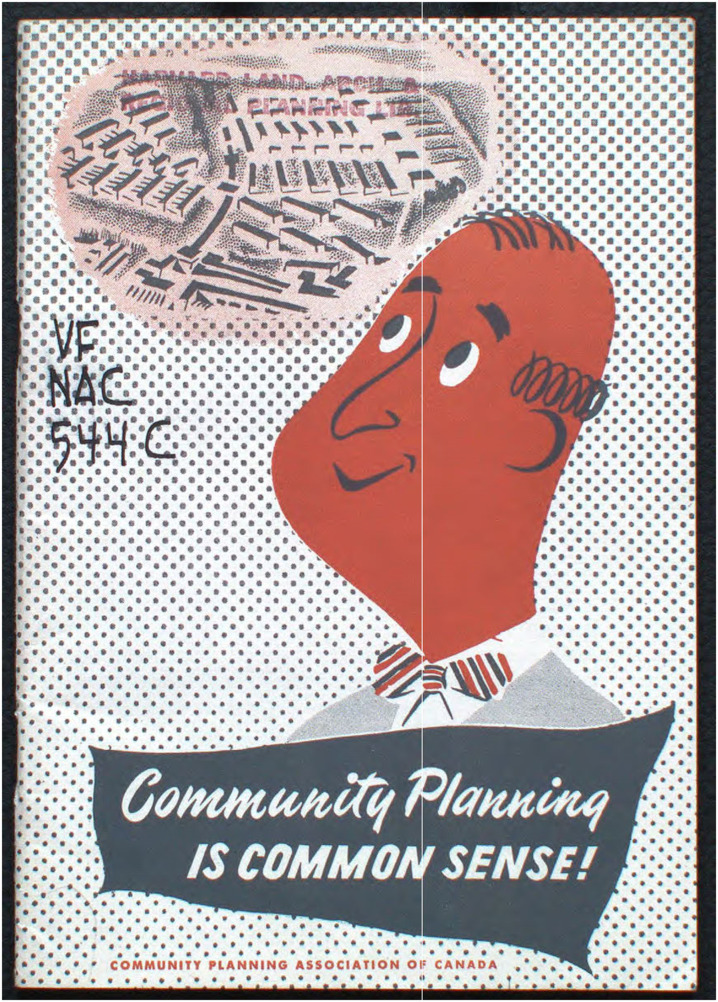
CPAC Advocacy Booklet, 1948. *Community Planning is Common Sense!* is a 40-page booklet published by the CPAC in 1948, containing explanations of the need for planning, descriptions of early citizen initiatives and lists of books and films. The thought bubble on the front cover is an image modelled on the Modernist design for a new satellite town proposed in the 1944 *Greater London Plan*. Thousands of copies of *Community Planning is Common Sense!* were sold by local CPAC branches across Canada at the highly discounted price of ten cents, or simply given away to interested citizens. Image source: Community Planning Association of Canada. *Community Planning is Common Sense!* Ottawa: the Association, 1948. Retrieved from Francis Loeb Library vertical files, Harvard University [NAC 544 C].

**Figure 4.**
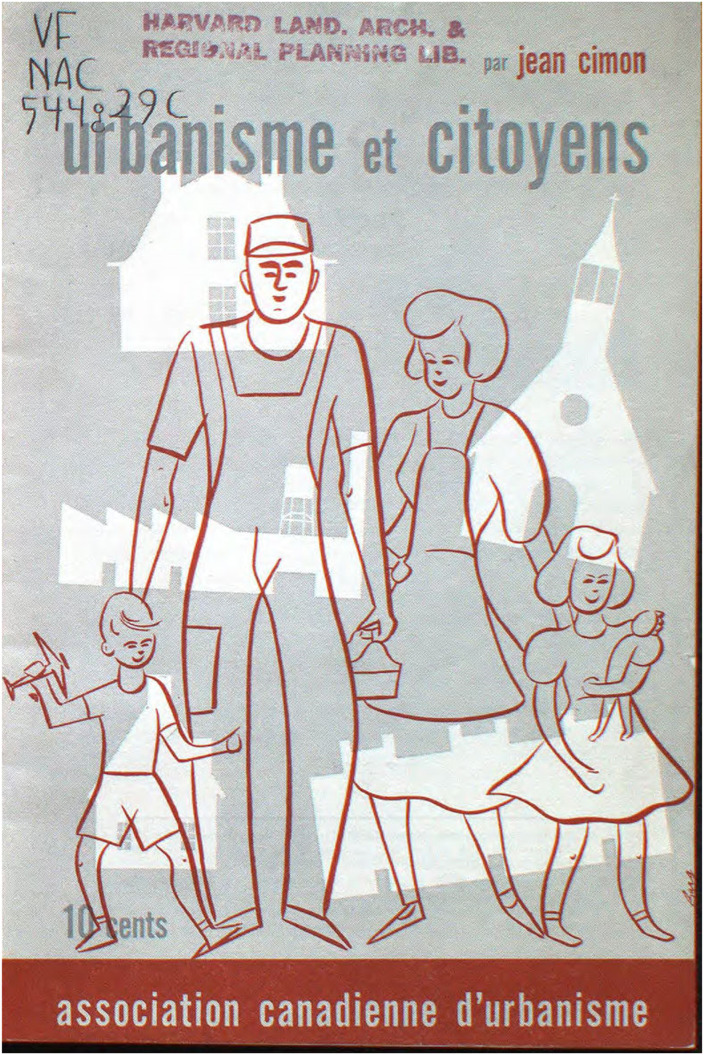
CPAC French language Advocacy Booklet, 1949. The CPAC hired a francophone community organizer, Jean Cimon, after some prodding from its Québec councillors. He wrote this 27-page booklet with some similar content to *Community Planning is Common Sense!* but a completely different cover. Instead of a businessman dreaming of a new community, we have a working-class family superimposed on outlines of a factory, a church and typical Québecois housing types. Source: Cimon, Jean. *Urbanisme et Citoyens*. Ottawa: l’Association Canadienne d’Urbanisme, 1949. Retrieved from Francis Loeb Library vertical files, Harvard University [NAC 544 29C].

**Figure 5.**
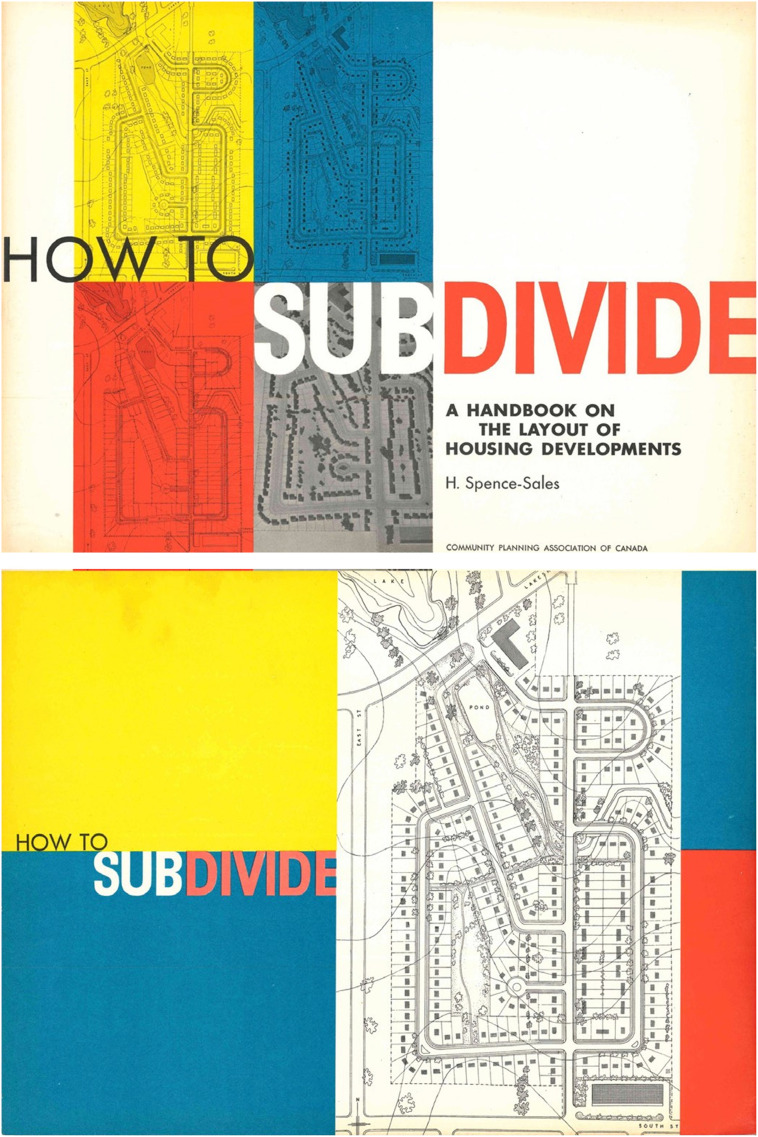
CPAC subdivision design manual, 1950. Few people knew how to design or build a modern subdivision in the post-war period, so CMHC funded a Summer Subdivision Design School in 1948 and 1949 conducted by McGill professor Harold Spence-Sales. CPAC published the school’s curriculum in a beautifully-designed 36-page monograph that was still in use decades later. Note that the front and back covers show the curvilinear street design with a cul-de-sac, which replaced the pre-war grid street system initially proposed for the site. Source: Spence-Sales, Harold. *How to Subdivide.* Ottawa: Community Planning Association of Canada, 1950. Author’s collection.

CPAC became the national clearing house for community planning, intended to educate the public and professionals about the purpose and benefits that community planning could afford to Canadian municipalities (Figures 3 and 4).^30^ Creating materials was necessary to educate both the public and professionals about what were considered good and bad planning practices following the collapse of planning in Canada from the Depression through the Second World War. CPAC in its early years frequently discussed plans of subdivision and their design, Clarence Perry’s Neighbourhood Unit, and the Nuclear City in their regular publications (*Layout for Living* and the *CPAC Newsletter*), in special publications (pamphlets and brochures), and in their conferences (Figure 5). CPAC’s promotion of ‘good’ subdivision design in the form of the Neighbourhood Unit, the Nuclear City and New Towns promoted suburban sprawl in Canada. Though their publications encouraged other forms of residential development, overwhelmingly, the topics encourage dispersed suburban growth in single-family homes.

The early goals of CPAC and CMHC from the Curtis Report directed this dispersed suburban growth: the need to build one million new homes in the next decade, for instance. CMHC provided funding for municipal servicing of lots and the construction of roadways on land for residential subdivisions, referred to as Land Assembly, through Section 35 of the *National Housing Act* (NHA). CPAC strongly promoted and closely followed this program. However, from the beginning, CPAC cautioned against focussing on the number of new homes as the Curtis Report does – Humphrey Carver’s article *Planning for Half a Million Houses* published in *Layout for Living* argues that the quality of the homes and their surrounding neighbourhoods is more important than the quantity of new units.^31^

**Figure 6.**
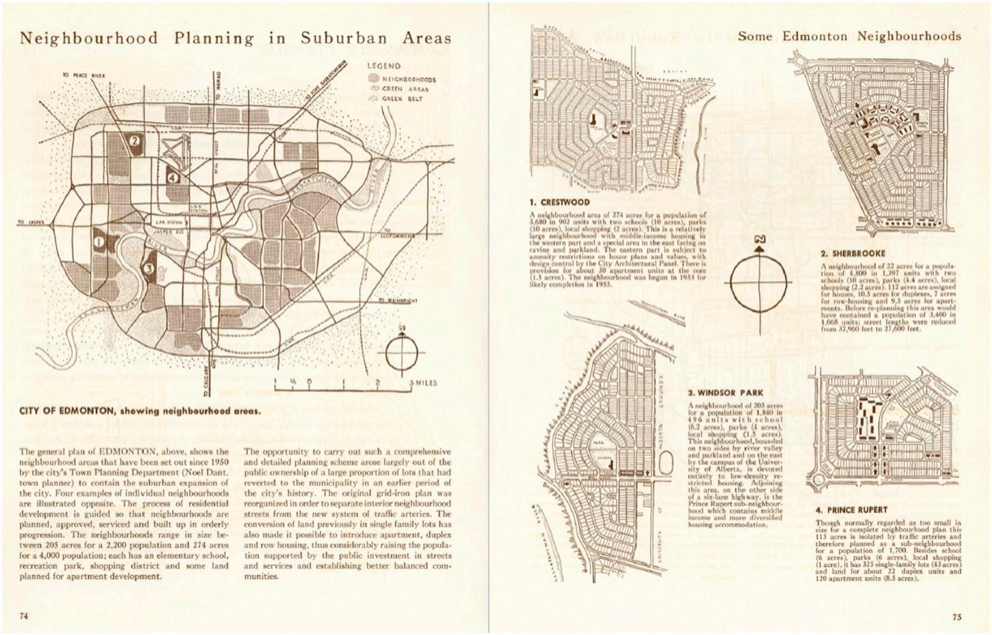
CMHC neighbourhood planning brochure distributed by CPAC, 1952–3. CMHC produced this 80-page housing design manual and distributed it in the CPAC’s *Community Planning Review* and the *Journal of the Royal Architectural Institute of Canada* in 1952 and 1953. The community planning chapter featured these Edmonton neighborhoods with curvilinear street designs and central schools, following Perry’s neighborhood unit concept. Source: CMHC, Housing Design Supplement Part II, Chapter 8 - Community Planning, *Journal of the Royal Architectural Institute of Canada,* vol 30 no 5, 74–75.

As with ‘good’ subdivision design, CPAC (and by association CMHC) influenced the types of planning practices that proliferated in Canada from the 1940s to the 1960s (Figure 6). CPAC and CMHC were joined at the hip in the first years after CPAC was formed in 1946. The CPAC National Council always included a CMHC representative on its roster – the longest serving representative was Humphrey Carver, from 1948 to 1957.

CPAC closely followed CMHC in the topics published in their early years. From 1946 to 1950, CPAC was governed nationally through an administrative office located at CMHC’s headquarters in Ottawa. The Secretary-Treasurer managed the administration and day-to-day activities of CPAC, assisted by staff loaned to CPAC from CMHC and served as the Editor of CPAC’s national periodicals. CPAC’s editorial team largely consisted of CMHC employees and made decisions about what planning types should be encouraged or discouraged in CPAC’s publications.^32^ By 1964, although CPAC had many publications, ‘much of its content is [sic] both repetitive and sketchy’ having frequently referenced the importance of community planning and effective subdivision design.^33^

Susan Briggs’ 1964 thesis created a detailed account of CPAC’s history to that year through 19 interviews^34^ with current and former executives and national office staff of CPAC. As a result, Briggs produced a detailed assessment of how CPAC operates as a pressure group.^35^ Briggs’ analysis of the relationship between CPAC and CMHC found that any negative repercussions effected by CPAC on CMHC were to be avoided. Any advertisement of the connection between CMHC and CPAC was discouraged from the 1950s onwards: CPAC no longer published details on funding sources, found other sources to supplement CMHC’s annual grant, and referred to the CMHC Representative as an Executive Councillor. To Briggs, these three factors meant that CPAC could ‘think and act more autonomously’; however, CPAC was still dependent^36^ on CMHC for the budget to support their staff, conferences and publications. Further, even though the persons interviewed by Briggs found that the CMHC Councillors ‘did not throw their weight about’, those interviewed unanimously agreed that CMHC Councillors such as Humphrey Carver, were ‘often the most “able,” “knowledgeable,” “well informed” and “most effective” men on Council’.^37^

## Planning Education

The Curtis Report emphasized the need for, and importance of effective planning, becoming a part of CPAC’s mandate. Throughout the 1950s, CPAC led discussions on ways to educate planning practitioners at Canadian universities using Canadian examples, following CPAC’s publication of *How to Subdivide* and its success. In CPAC’s quest to encourage community planning across Canada, it lobbied the federal government for funding for university programs to educate professional planners. In 1952, CMHC scholarships were announced through CPAC and advertised annually thereafter in the *CPAC Newsletter* and *Community Planning News*:

*It has been the privilege of the Association to help announce the terms of assisted study at the four schools of planning* [University of Toronto, Manitoba, British Columbia, McGill University], *to confer with those in charge of the courses upon the work they are doing, and to be associated with the publication and display of some of their studies and achievements*.^38^

In 1944, there were perhaps five part-time, practicing planning professionals in Canada.^39^ Carver’s article in *Community Planning Review* estimates that by 1954, 43 students had graduated from Canadian university courses on planning. Of those graduates, 39 received funding from CMHC’s scholarships, and 33 pursued careers in community planning in Canada. These initial figures show that, from 1947 to 1954, Canada produced approximately 5 professional planners per year. Within 10 years of the publication of the Curtis Report, which had stressed the great need for and importance of community planning by qualified professionals, Canadian universities were still only producing annually the tiny number of professional planners practicing in Canada at the end of the war.

## Better Enabling Legislation

Prior to 1944, community planning in Canada was rare, and where plans and planning professionals existed, these plans were advisory in nature.^40^ The Curtis Report describes that, while ‘most provinces have passed statutes, and town planning powers of a kind have been available to local authorities’, existing legislation is too general, ‘represents a form of negative control’, and would be insufficient even with substantial changes.^41^ Further, by 1944, ‘town planning legislation in Canada had [*sic*] not been successful; and for the most part it was [*sic*] inoperative’.^42^ At a national level, the Curtis Report recommended that a federal agency dedicated and ‘equipped effectively’ should be able to ‘encourage and assist the provinces in passing the necessary enabling legislation for municipal and regional planning’.^43^ CMHC, as the federal government agency, was tasked with creating and funding CPAC to follow through on many of these ideas presented by the Curtis Report.

As previously discussed, Harold Spence-Sales’ *How To Subdivide* can be seen as the study of minimum planning standards (at least for residential areas) recommended by the Curtis Report. Spence-Sales produced the other recommended report, a study on model planning legislation, for CMHC in 1949, with assistance from Norah McMurray.^44^ In this report, Spence-Sales investigates the number of town planning agencies and plans in existence; planning activity is found to be limited to the major urban centres for each province.

McMurray revisited the topic of active planning legislation and planning administration across the provinces in Canada in 1952, publishing the study through CPAC for a wider audience as *Outlines of Canadian Planning Law*.^45^ McMurray describes for each province (except Quebec and Newfoundland) the active planning legislation and the duties of the planning administration. At the same time McMurray’s study was published, the CPAC National Office and Provincial Divisions submitted briefs arguing for better planning and better enabling legislation. Comparing J. B. Milner’s *Community Planning: A Casebook on Law and Administration* in 1963 to Norah McMurray’s report in 1952 reveals that changes to planning legislation occurred in several Canadian provinces in 1955 and 1960. In Ontario and British Columbia, CPAC’s lobbying encouraged site plan and subdivision controls, and discouraged ribbon development, respectively. In both cases, CPAC’s lobbying created greater public support for establishing municipal planning commissions and hiring qualified planning professionals across Canada.

## Conclusion

By 1960, CPAC had accomplished its goals of public education on the topic of community planning through its widespread publications and the advocacy efforts of its more than 4000 members. CPAC had sponsored no less than 15 national conferences and dozens more at the regional and local scale; representatives of CPAC spoke to municipal and provincial officials, planners, architects, engineers, and advocacy groups about the importance of community planning. Members of CPAC taught community planning at four universities and organized at least five extension courses to educate working professionals about the intricacies of community planning law, practice, and administration among other topics. From its national office, CPAC had published 4 periodicals (with hundreds of issues), 3 monographs, 11 special pamphlets, and even more conference proceedings, mimeographs, brochures, and reports. The public had been educated on the benefits of community planning, which was firmly supported by municipal planning bodies and provincial planning legislation in most of Canada by 1963.^46^ Now that the public had been convinced of the benefits of community planning, what was left for CPAC to accomplish?

For the remainder of its life, CPAC would frequently re-evaluate its purpose: in 1954, in 1967, and throughout the 1970s in order to ensure continued funding from CMHC. In the early 1970s, CPAC attempted to re-focus as a community advocacy group opposing CMHC’s urban renewal programs. Not surprisingly, CMHC revoked all funding under this objective, which led to CPAC’s national collapse in 1978.^47^ The CPAC would carry on in some provinces and municipalities after the national office collapsed with the support of volunteers and local staff and is still active in Alberta today.^48^ Despite its unfortunate conclusion, CPAC’s early activities, actors and interests strongly influenced the development of planning in post-war Canada, shaping the conditions for lasting impacts on the Canadian landscape.

This research has revealed that CPAC influenced nearly every aspect of the post-war planning supply chain in Canada: it generated demand for planning and for qualified professionals, supplied educational materials to the public, students, and professionals, and financed training and research in planning. However, CPAC, while effective and influential in the development of Canadian planning in the immediate post-war period, was ***not*** the central institution responsible for the path dependent suburbanization of Canada. As noted above, there are four required elements to determine critical junctures and path dependency: the intentional usage of a particular moment in history as a critical juncture; a positive feedback effects loop instigating a specific, long-standing pattern of development; the creation of a system which prioritizes certain actors/opportunities and their resultant effects on the overall structure being created; and the Co-evolutionary processes of development between the instigating actors and their partners in maintaining the chosen system.

The second, third, and fourth points are discernable from the actions noted above. The CPAC assisted in creating a positive feedback loop capitalizing on market demand for homes in planned communities. CPAC both encouraged demand for and supplied the methods (*How to Subdivide)* and means (professional planners) to achieve new community designs for suburban development using curvilinear road layouts and the neighbourhood unit. These methods and design standards may have changed, but the demand for suburban development persists in Canada today. Likewise, CPAC advocated for mandatory planning legislation including subdivision controls while also developing subdivision guidelines, thereby creating a system that prioritizes low-density suburban development of a particular kind. CPAC and CMHC maintained and evolved the chosen system of supplying professional planners, researching best practices for subdivision design and providing funds for both.

However, the first point, requiring the intentional usage of a moment in history as a critical juncture was not found in our analysis of CPAC. Though the Curtis Report appears to be the critical juncture, CMHC brought CPAC to life by acting on the Curtis Report’s recommendations. CPAC’s primary funding body – CMHC – requires further examination as the possible central institution responsible for the path dependent suburbanization of Canada.

This article extends Harris’ depiction of CMHC as the central actor in the creeping conformity of Canadian suburbanization by re-examining the processes through an HI lens and demonstration the nature of CPAC’s contribution. CMHC also played a major role in shaping the planning of Canadian suburbs in the post-war period by creating new suburban design standards and reviving community planning through its influence over CPAC. CMHC facilitated a major change in Canadian urban structure, even though urban planning and development are within provincial and municipal jurisdictions. CMHC used a multi-pronged approach to promote the federal policy agenda, including advocacy, education, research, national standards, capacity-building, and demonstration projects across Canada.^49^ CMHC facilitated widespread, top-down advocacy for community planning through CPAC in the 1940s and 1950s, but withdrew its funding in the 1970s, when the CPAC’s advocacy turned against CMHC’s urban renewal programs.

CMHC’s role in changing suburban community planning will be analyzed in future research. Although our research demonstrates that CPAC was ***not*** the central institution in Canadian acceptance of the role of community planning in Canada, its legacy lives on through the provincial legislation that the Association fostered in the post-war era. In 2023, every Canadian municipality – from the smallest village in Nunavut to the largest city – Toronto – had a comprehensive land use plan and zoning bylaw that were statutory legislation guiding future development.^50^

## Notes

1. C. A. Curtis. “Housing and Community Planning.” Final Reports of the Subcommittee on Housing and Community Planning for the Canada Advisory Committee on Reconstruction (“Curtis Report”) (Ottawa ON: King’s Printer, 1944).

2. “Community planning” is a term used mainly in Canada to describe professional activity generally known as town planning in Britain, city planning or urban planning in the United States, or urbanisme in France. Widespread adoption of this term came only after the 1944 publication of the Curtis Report.

3. American Society of Planning Officials, Planning: selected papers from the Annual Planning Conference of the American Society of Planning Officials meeting jointly with the Community Planning Association of Canada, September 25–29. 1955. (Chicago: ASPO, 1955).

4. American Society of Planning Officials, Planning: selected papers from the Annual Planning Conference of the American Society of Planning Officials meeting jointly with the Community Planning Association of Canada, Toronto 1965. (Chicago: ASPO, 1965). Ottawa planner Eric Thrift, a CPAC founder and former Vice President, was President of ASPO in 1964–65. See Eric Thrift Fonds, Queen’s University Archives.

5. Sue Hendler and Julia Markovich. *I Was the Only Woman: Women and Planning in Canada* (Vancouver: UBC Press, 2017) analyses how CPAC was less misogynistic than the Town Planning Institute of Canada.

6. Richard Harris, *Creeping Conformity: How Canada Became Suburban, 1900-1960* (Toronto: University of Toronto Press, 2004).

7. Pierre Filion, “Suburban Inertia: The Entrenchment of Dispersed Suburbanism,” *International Journal of Urban and Regional Research* vol. 39 (2015): 633–634.

8. Harris, *Creeping Conformity*.

9. Jill Grant, Pierre Filion and Scott Low, “Path dependencies affecting suburban density, mix, and diversity in Halifax,” *The Canadian Geographer/Le Geographe Canadien* vol. 63 (2019): 250.

10. Harris, *Creeping Conformity*.

11. Paul Pierson, “Increasing Returns, Path Dependence, and the Study of Politics,” *The American Political Science Review* vol. 94 no. 2 (2000): 251–267; Andre Sorensen, “Taking path dependence seriously: an historical institutionalist research agenda in planning history,” *Planning Perspectives* vol. 30 (2015):17–38.

12. Andre Sorensen and Paul Hess, “Building suburbs, Toronto-style: Land development regimes, institutions, critical junctures and path dependence,” *Town Planning Review* vol. 86 no. 4 (2015): 411–437; John Sewell, *The Shape of the City: Toronto’s Struggles with Modern Planning* (Toronto: University of Toronto Press, 1993); John Sewell, *The Shape of the Suburbs: Understanding Toronto’s Sprawl* (Toronto: University of Toronto Press, 2009).

13. Sorensen, “Taking path dependence seriously”; Sorensen and Hess, “Building suburbs, Toronto-style”.

14. Giovanni Capoccia, “Critical Junctures,” in Fioretos, O., Falleti, T., and Sheingate, A, (eds.) *The Oxford Handbook of Historical Institutionalism* (Oxford: Oxford University Press, 2016).

15. Sorensen, “Taking path dependence seriously”; Giovanni Capoccia and Daniel Kelemen, “The Study of Critical Junctures: Theory, Narrative, and Counterfactuals in Historical Institutionalism,” *World Politics* vol. 59 (2007): 341–69; Andre Sorensen, “Taking critical junctures seriously: theory and method for causal analysis of rapid institutional change,” *Planning Perspectives* (November 2022; early release), DOI: 10.1080/02665433.2022.2137840

16. Andre Sorensen, “Planning History and Theory: Institutions, Comparison, and Temporal Processes,” in C. Hein (ed.), *Routledge Handbook of Planning History*. (London, New York: Routledge, 2018), p. 43.

17. Sorensen, “Planning History and Theory,” p. 38;

18. Filion, “Suburban inertia”; Grant, Filion and Low, “Path dependencies”; Sorensen and Hess, “Building suburbs, Toronto-style”.

19. Andre Sorensen, “Global Suburbanization in Planning History,” in C. Hein (ed.), *Planning History Handbook* (London: Routledge, 2017): 386–399; Sorensen, “Taking path dependence seriously”.

20. Sorensen, “Global Suburbanization in Planning History,” 396.

21. Sorensen, “Global Suburbanization in Planning History.”

22. Harris, *Creeping Conformity*; John Bacher, *Keeping to the marketplace: The evolution of Canadian housing policy*. (Montreal & Kingston: McGill-Queen's University Press, 1993).

23. Clifford Austin Curtis (1899-1981) was a professor of economics at Queen’s University, with a secondary interest in local government and community planning. He was first elected as an Alderman of the City of Kingston in 1936; founded its Town Planning Commission in 1937; authored its first zoning bylaw in 1941 and was subsequently Mayor of Kingston from 1948 to 52.

24. Humphrey Carver, *Compassionate Landscape* (Toronto ON: University of Toronto Press, 1975); Carver was a Vice-President of the CPAC before being recruited to the CMHC in 1948 as its research coordinator, giving him a central position in CMHC’s post-war planning activities.

25. The Community Planning Association of Canada fond, which dates between 1946 and 1978, included 3.1 m of textual records, including background materials, treasurers’ records, financial records and other files, logged under the archival reference number R2852-0-9-E or MG28 I 14.

26. The CMHC Fond at LAC (reference number R1197-0-4-E) includes a variety of materials ranging from textual records to architectural drawings, maps, technical drawings, film reels and audio cassettes, according to LAC finding aid.

27. Harold Spence-Sales, *How to Subdivide: A handbook on the layout of housing developments* (Ottawa, ON: Community Planning Association of Canada, 1950).

28. Periodicals from the National Office of the CPAC: *Layout for Living* (1947-1950), *CPAC Newsletter* (1950-1954), *Community Planning News* (1954-1959), and *Community Planning Review* (1951-1971).

29. Special publications such as Harold Spence-Sales, *How to Subdivide*; Norah McMurray and Harold Spence-Sales, *Outlines of Canadian Planning Law: A Comparative Survey of Town Planning Legislation Operative in the Provinces of Canada* (Ottawa: Community Planning Association of Canada, 1952); *Community Planning is Common Sense* (Ottawa: Community Planning Association of Canada, 1948); and *A Case for Satellite Towns* (Ottawa: Community Planning Association of Canada, 1953).

30. Curtis, “Housing and Community Planning,” 169–170.

31. *Layout for Living* 1947 no. 2, p. 2.

32. Alan H. Armstrong was the first secretary of the CPAC and editor of *Layout for Living*, selecting the topics for publication while operating out of his office at CMHC Headquarters in Ottawa while on loan to the CPAC from his position as Chief Planner.

33. Susan J. Briggs, *The Community Planning Association of Canada: A Pressure Group* (Undergraduate Thesis: Queen’s University, 1964), p. 1. Retrieved from LAC MG28 I 14 (R2852-0-9-E). CPAC 8, File 15. Sketchy likely meaning vague in this context.

34. Persons interviewed by Briggs: Alan H. Armstrong, Eric Beecroft, Major-General M.L. Brennan, J.F. Brown, Humphrey Carver, T. S. Chutter, W. Harold Clark, Catherine Corbett, W. A. Dempsey, Mrs. H. A. Elliot, Jennifer Joynes, Miss J. M. Laventure, A. L. S. Nash, John Pearson, Norman Pearson, Mrs. R. H. Scrivener, D. Taylor, Eric Thrift, and E. F. Tonge.

35. Briggs defines “pressure group” as “all groups which collectively pursue common political goals (Excluding parties, that is groups which seek directly to govern)” as quoted in Harry Eckstein, *Pressure Group Politics* (London: George Allen & Unwin, 1960), p. 9.

36. Both Briggs and financial statements from the CPAC fonds show that the CMHC grant accounted for at least 76% of the annual CPAC’s revenue (both in 1959 and 1963).

37. Briggs, *CPAC*, p. 42.

38. CPAC *Newsletter* 1952 no. 5, p. 5.

39. Humphrey Carver, “The Universities and Community Planning,” *Community Planning Review* vol. 4 (1954): 19–23.

40. Curtis, *Curtis Report*, 168–169.

41. Curtis, *Curtis Report*, 169.

42. Curtis, *Curtis Report*, 169.

43. Curtis, *Curtis Report*, 169–170.

44. Harold Spence-Sales, *Planning Legislation in Canada* (Ottawa: CMHC, 1949).

45. McMurray, *Outlines of Canadian Planning Law*: ii.

46. Quebec and New Brunswick are exceptions.

47. LAC MG28 I 14 (R2852-0-9-E), CPAC 4, File 5, *Minutes – Special Meeting of Council, CPAC Thinking Conference*.

48. Today, the CPAC’s former Alberta Division operates as the Community Planning Association of Alberta. See: https://www.cpaa.biz/content/about-0, Accessed November 2022. The Community Planning Association of Canada, Nova Scotia Division fonds can be found at: MS-11-3, Dalhousie University Archives, Halifax, Nova Scotia; The British Columbia Division of the CPAC (1947–1977) fonds are at the City of Vancouver Archives, AM683. The archives of the Quebec Division are lost; its activities were reconstructed by Vincent Desforges in *The Development of Modern Urban Planning in Quebec between 1946-1973*, Master’s Report, School of Urban and Regional Planning, (Kingston ON: Queen’s University, 2019).

49. Carver, *Compassionate Landscape; Harris, Creeping Conformity*.

50. Vancouver is a partial exception since it has only partially updated its Official Community Plan since the 1950s.

